# Beyond Star Trek: Latin American science fiction and the ELSI of emerging technologies

**DOI:** 10.3389/fsoc.2026.1898512

**Published:** 2026-10-29

**Authors:** Cristián Londoño-Proaño

**Affiliations:** Centro de investigaciones de Ciencias Humanas y de Educación (CICHE), Universidad Tecnológica Indoamérica, Ambato, Ecuador

**Keywords:** algorithmic sovereignty, anticipatory governance, cloning, ELSI, epistemic justice, eugenics, genetic engineering, global south

## Abstract

Science fiction has been recognized as a place of anticipatory governance, with audiovisual productions such as Star Trek serving as prominent references in ELSI’s research on genetics, biotechnology, and human enhancement. Latin American science fiction has developed parallel and largely unrecognized imaginaries involving genetics, computing, and biotechnology from structurally distinct social positions rooted in inequality, colonial erasure, and the historically expendable body. This paper aims to demonstrate that Latin American speculative fiction constitutes an underexplored form of anticipatory governance of the Global South, one whose significance lies not in having anticipated ELSI’s core debates, but in framing them from a different standpoint: that of the communities on whom the risks of genetic engineering, algorithmic systems, and human enhancement are most likely to fall. Using a qualitative methodology that combines comparative narrative analysis and self-reflexive autoethnography, three case studies are examined: The Unproductive, which constructs a dystopian society based on human cloning and genetic stratification that anticipates contemporary debates on eugenics and corporate biotechnology; Quipucamayoc, which reimagines Andean information systems as protocomputational knowledge, anticipating issues of algorithmic sovereignty and epistemic justice; and El Yachak, which proposes an organic biotechnology incorporated rooted in indigenous epistemology as a countermodel to reductive enhancement frameworks. The analysis reveals that these narratives encode ELSI concerns through the sociological realities of structural marginalization, demonstrating that anticipatory governance is not a monopoly of northern science fiction. This article proposes a pluriversal framework for expanding research on ELSI and anticipatory governance studies beyond the Anglo-American canon, with direct implications for inclusive and culturally situated policy design in an era of growing genetic and biotechnological capacity.

## 1 Introduction

Science fiction has long functioned as a distributed space of ethical reasoning. It can be considered as a cultural laboratory in which societies test, question, and imagine the implications of technologies before these technologies are fully materialized (Londoño-Proaño, 2023). As Greenbaum (2025) argues, enduring cultural narratives about technology, passed down from generation to generation and embedded in collective memory, shape how societies interpret and respond to emerging innovations such as artificial intelligence, genetic engineering, and neurotechnology, helping to shape regulatory discourse by embedding philosophical assumptions and ethical norms into governance frameworks. Audiovisual productions such as Star Trek have occupied a prominent place in this tradition. For more than six decades, some of the episodes have addressed genetic enhancement, cloning, artificial intelligence, and the limits of human enhancement have served as prominent references in ELSI research, shaping public expectations about biomedical innovation, informing policy debates, and generating the sociotechnical imaginaries within which the actual governance of emerging technologies operates (Greenbaum, 2025).

Latin American science fiction, and its Andean neo-indigenist current in particular, has developed a parallel corpus of speculative narratives that use the same technologies but from structurally different social positions as positions shaped by the lived realities of colonial dispossession, structural inequality, and bodies historically marked as expendable (López-Pellisa, 2025; Nahar et al., 2025). Prado Sejas (2023) explains that neo-indigenist narratives place indigenous subjects at the center of complex technological futures, challenging Western hegemonic visions of progress. This has generated its own ideas of anticipatory governance, or governance that looks toward the socio-political changes that new technologies may bring about in the future, and acts in the present to anticipate these especially damaging effects.

This article hypothesizes that Latin American speculative fiction constitutes a distinct and little-explored form of anticipatory governance developed in the Global South, whose contribution lies not in chronological priority but in the standpoint from which it frames ELSI concerns: that of communities historically positioned as the objects, rather than the beneficiaries, of technological governance. This hypothesis can be tested through a systematic comparative narrative analysis of the ELSI concerns encoded in these texts, examining how their framing of genetic engineering, algorithmic systems, and human enhancement differs from that of the Anglo-American tradition represented by Star Trek.

To test this hypothesis, three case studies are examined: The Unproductive (Londoño-Proaño, 2014), a dystopian novel that builds a society founded on human cloning and genetic stratification; Quipucamayoc (Salvo, 2014), a short story that reimagines Andean information systems as protocomputational knowledge; and El Yachak (Páez, 2007), a story that proposes an embodied organic biotechnology based on indigenous epistemology. Each text addresses a distinct axis of emerging technology: genetic engineering, algorithmic governance, and biotech enhancement, and each codifies ELSI concerns that conventional governance research has not yet fully integrated.

## 2 Theoretical framework

### 2.1 Anticipatory governance and science fiction

Anticipatory governance, as theorized by Guston (2014), refers to a broad societal capacity to envision, interact with, and integrate emerging knowledge-based technologies while governance remains meaningful and possible. Anticipatory governace motivates activities designed to develop foresight, stakeholder engagement, and reflective integration capabilities before technology lock-in occurs. As Barben et al. (2008) elaborate in their fundamental treatment of anticipatory governance in the context of nanotechnology, this capacity operates through three interconnected modes: (1) forecasting, which is related to the anticipation and evaluation of emerging technologies; (2) participation, which is related to public participation in the deliberation on socio-technical futures; (3) integration, which is related to the incorporation of broader societal considerations into research and development contexts. Crucially, anticipatory governance does not presuppose predictive certainty: rather, it implies an awareness of the co-production of sociotechnical knowledge and the importance of imagining rich sociotechnical alternatives that can inspire its responsible use.

Science fiction participates in this function of governance not as a prediction, but as a cultural essay. Speculative narratives generate what Jasanoff and Kim (2015) call sociotechnical imaginaries, that is, collectively held and institutionally stabilized visions of desirable futures and the social orders they presuppose. From a visual perspective of culture and communication, science fiction audiovisual narratives function in particular as what Londoño-Proaño (2026a), calls imagination triggers, symbolic devices that stimulate creative thinking, guide design expectations, and mediate technological meanings before those technologies materialize. Rather than establishing direct causal relationships between fiction and technical development, imagination triggers contribute to the formation of shared imaginaries that influence how societies anticipate and evaluate emerging technologies. This symbolic and communicative dimension of anticipatory governance, which operates through narrative, image, and design rather than formal regulatory mechanisms, constitutes precisely the analytical space that this article seeks to expand into the Global South.

These imaginaries shape research agendas, public trust, risk perception, and regulatory frameworks through concrete discursive mechanisms rather than abstract influences. Johnson (2011) identifies this dynamic as science fiction prototyping, in which fictional artifacts function as exploratory models that channel the attention of science and engineering toward specific technological trajectories. This channeling effect is not merely theoretical. Londoño-Proaño (2026a), documents that Martin Cooper explicitly attributed the Star Trek communicator device with the direct inspiration for developing the first portable mobile phone, and that the Qualcomm Tricorder XPrize was explicitly modeled after the series’ diagnostic device. These examples are concrete. A fictional depiction shaped the research agendas of working engineers decades later.

The same mechanism operates on public trust and technological adoption. When Cisco CEO John Chambers introduced telepresence video conferencing technology in 2006, he explicitly compared it to Star Trek’s teleportation to build public trust in an unknown communication paradigm (Londoño-Proaño, 2026a), illustrating how narrative familiarity, established decades earlier through fiction, can be strategically invoked to reduce the perceived novelty and risk of an emerging technology at the time of its Market introduction.

At the level of design expectations and interaction paradigms, Axtell and Munteanu (2021), as discussed in Londoño-Proaño (2026a), found that voice UI designers explicitly used the idealized human-computer interactions imagined in Star Trek: The Next Generation as a reference standard for evaluating and improving contemporary systems, demonstrating that speculative imaginaries can directly shape the technical criteria that engineers apply to the design of real interaction.

Finally, at the level of alternative technological epistemologies, Andean speculative fiction encodes forms of technological knowledge rooted in organic, relational, and community traditions, quite distinct from the instrumental rationality embedded in dominant Western technological paradigms (Londoño-Proaño and Endara, 2024). This corpus does not directly address regulatory frameworks, but its documented alternative epistemologies constitute exactly the kind of underrepresented conceptual resource that this article argues ELSI research should use to govern genetic and biotechnology innovation in culturally diverse contexts.

Audiovisual productions such as Star Trek have been widely analyzed as artifacts of anticipatory governance that shaped public expectations about genetic enhancement, artificial intelligence, and the limits of human identity (Greely, 2023). Star Trek’s Augments, genetically modified humans destabilizing the Federation’s social order, anticipate contemporary debates about CRISPR, polygenic risk scoring, and the resurgence of eugenic logics under a biomedical framework (Vongehr, 2020; Hogan and Decker, 2021). As the cases documented above illustrate, this influence operates not through abstract cultural resonance, but through traceable pathways that connect specific narrative representations to concrete decisions in engineering research, product design, and public communication.

The use of science fiction as an analytical lens for ELSI has been actively developed by Greenbaum (2022), who argues that audiovisual science fiction, specifically the anthology series Black Mirror, provides students and scholars with an optimal framework for examining the ethical, legal, and social implications of emerging technologies, precisely because its dystopian narratives situate technological concerns within recognizable social realities in front of our current timeline. Critically, Greenbaum (2022) identifies the need to make ELSI education and analysis international, inviting students and perspectives from institutions around the world to develop more comprehensive analyses of the technological implications from their respective social and academic contexts. This agenda has been more recently and explicitly expanded by Greenbaum (2025), who advocates for a systematic investigation of how cultural narratives, from Frankenstein to Astro Boy, shape the ethics of technological governance across different cultural traditions. This article responds directly to both contributions: if Black Mirror and Star Trek serve as legitimate analytical corpus of ELSI within the Anglo-American tradition, Latin American science fiction constitutes an equally legitimate, and structurally different, corpus with respect to the Global South, one that situates the same technological concerns within social realities defined not by proximity to Silicon Valley or by the Western and Eastern cultural frameworks of Asia, but also by colonial history, structural inequality and the indigenous epistemologies of the Andean world.

On the other hand, science fiction’s anticipatory governance function is not culturally uniform, because where speculative narratives emerge from contexts of structural inequality, colonial history, and marginalized epistemologies, they construct fundamentally different problematic governance spaces. They do not just ask, what if we could modify human genes? But *who* will be designated as genetically deficient? Not only, what if computers could store all knowledge? But *whose* knowledge will be stored and *whose* knowledge will be erased? These are questions that Latin American speculative fiction has been asking for decades and that ELSI’s research has not yet systematically incorporated.

### 2.2 ELSI scholarship and the global south

ELSI research originated in the Human Genome Project’s ethical review frameworks. As Greenbaum (2013) documents, the field was born out of a last-minute revision of a 1988 speech by James Watson, in which Watson allocated 3 % of the Human Genome Project’s budget (Cook-Deegan, 1994) specifically for the study of ethical, legal, and social implications as his first act as director of the NIH’s Human Genome Institute. In the decades that followed, hundreds of millions of public dollars were distributed under this mandate, establishing ELSI as a building block of genomics research. Crucially, Greenbaum (2013) himself identifies a central limitation of this framework: ELSI needs to be more international to reflect the international nature of biomedical research, recognizing that its diffusion across borders is a non-trivial problem given the marked differences in society, morality, and law across the planet. This article responds directly to that challenge by incorporating Latin American speculative fiction into the conversation about ELSI, a corpus that has generated ideas of anticipatory governance precisely from those social and moral positions that the Anglo-American tradition of ELSI has not yet integrated.

In the realm of biotechnology governance, this focus means that the ethical concerns of communities whose bodies have historically been subjected to non-consensual experimentation, eugenic policies, and biomedical exploitation are structurally underrepresented in the frameworks governing the technologies most likely to affect them. Latin American science fiction, particularly its Andean neo-indigenist current, offers a partial correction: by narrating the encounter between emerging technologies and bodies marked by colonial history, these texts represent a form of anticipatory governance that is at once political, epistemic, and ethical.

The ELSI epistemic approach has been questioned by the academic field of Latin American science fiction itself. Londoño-Proaño and Endara (2024) have argued that Andean speculative fiction encodes technological, organic, incorporated, relational forms of knowledge that fall outside the parameters of Western ELSI frameworks, precisely because they arise from epistemological traditions that those frameworks do not recognize as technological.

### 2.3 The three primary texts

The three texts examined in this article represent distinct nodes within the Latin American neo-indigenist tradition of science fiction, each addressing a different technological axis.

The Unproductive (Londoño-Proaño, 2014) is an Ecuadorian dystopian novel, now in its third edition. Cristián Londoño-Proñao is a renowned science fiction writer and researcher (Endara, 2024). The novel is set in the twenty-second century. Its central concept, a global corporate order based on human cloning, genetic stratification, and the systematic elimination of the “unproductive,” places it in direct dialogue with contemporary debates about germ editing, corporate biotechnology, and the resurgence of eugenic logics. The novel was written in the immediate aftermath of Dolly’s cloning (1996) and the completion of the Human Genome Project (2001), reflecting a deliberate commitment to what these milestones implied for social organization. It has received sustained critical attention: Rodrigo-Mendizábal (2014) analyzed it through Jameson, Heidegger and Maturana as a critique of the corporate-genetic model, arguing that it constitutes an extrapolation of the tendencies of globalized capitalism toward a dehumanized future; more recently, Endara (2025) situates the novel in contemporary debates about artificial intelligence and human-machine coevolution, reading it not as a dystopia but as an anti-utopia, a society that takes the current logic of productivity to its ultimate consequences, serving the benefit of a few bio/techno/entrepreneurs sustained by the work of the majority. The novel also appears both in Historia de la ciencia ficción latinoamericana II edited by López-Pellisa and Ares (2021) and in La ciencia ficción en América Latina edited by Kurlat Ares and De Rosso (2021), confirming its place within the continental academic canon.

Quipucamayoc (Salvo, 2014) is a Peruvian short story published in the collection The First Peruvian in Space. Daniel Salvo is considered one of the main cultivators of the genre in Peru (López-Pellisa and Ares, 2021). The story analyzed has been described as one of Salvo’s most solid and recognized narratives, a story that exemplifies the insertion of a contemporary computer virus, transposed to the sixteenth century, through a quipucamayoc who introduces an erroneous sequence between the infinite knots of a quipu (López-Pellisa, 2025). This rivalry between a subject of the empire and the Tawantinsuyu generates a catastrophic information crisis with consequences at the systemic level, turning it into an accurate anticipated narrative of algorithmic failure, data governance, and epistemic sovereignty.

El Yachak (Páez, 2007) is an Ecuadorian short story published in the collection Deep in the Galaxy (Casa de la Cultura Ecuatoriana, 1994; reissued by Alfaguara, 2007). Santiago Páez Gallegos is recognized as a key author of contemporary Ecuadorian science fiction, whose narrative is characterized by exploring the relationship between anthropology and literature, working on indigenous motifs in relation to space travel and the search by extraterrestrial beings for ancestral medicines (López-Pellisa, 2015). The story narrates a first-contact scenario between José Sánchez, an elderly Kichwa healer in the Andean highlands near the Peguche waterfall, and an alien ship whose crew has been affected by *espanto*, the Andean illness of soul-fright. The healing of the yachak from the alien ship through ritual and embodied knowledge constitutes a narrative model of indigenous biotechnology that challenges dominant frameworks of enhancement and anticipates contemporary debates about the governance and appropriation of non-Western biological knowledge.

It should be noted that both Cristián Londoño-Proaño, Daniel Salvo and Santigo Páez are considered in the Latin American History of science fiction (Kurlat Ares and De Rosso, 2021; López-Pellisa and Ares, 2021).

## 3 Methodology

This article employs a qualitative methodology that combines two complementary approaches: comparative narrative analysis and reflexive autoethnography.

### 3.1 Comparative narrative analysis

Comparative narrative analysis is applied to all three texts to identify and map the ELSI concerns they encode. The analysis follows three thematic axes established *a priori* based on the focus of the Research Theme on emerging technologies: (1) genetic engineering and biotechnology, including cloning, genetic stratification and enhancement; (2) computational and algorithmic systems, including data governance, epistemic sovereignty, and systemic failure; and (3) human enhancement frameworks, including the ethics of intervention in biological systems and the governance of non-Western knowledge.

For each text, the analysis proceeds with identifying the emerging technologies narrated; characterizing the social actors, institutions, and power relations through which those technologies operate; extracting the ethical, legal, and social concerns explicitly or implicitly codified in the narrative; and establishing correspondences between those concerns and current debates about ELSI governance. This approach is based on established practices in science fiction studies (Rieder, 2017) and the sociology of scientific imaginaries (Jasanoff and Kim, 2015).

Because two of the three texts have not been translated into English, the analysis incorporates extended excerpts from the primary texts, treated in the way social scientists incorporate interview transcripts or ethnographic field notes: as primary data that allow readers to assess each interpretation against the material it interprets. Excerpts are presented in the author’s English translation, and the original Spanish passages are provided in the Supplementary material. Excerpts from *The Unproductive* are quoted from the third edition of the novel (Londoño-Proaño, 2025b).

### 3.2 Author-reflexive autoethnography

The analysis of The Unproductive employs an additional methodological layer: self-reflexive autoethnography, in which the researcher-author examines his or her own creative work as a place of knowledge production (Orel, 2024). This approach is methodologically justified in STS and ELSI contexts for two reasons.

First, the novel’s intentional narrative architecture, the specific concerns of ELSI that motivated certain design choices, is accessible only through thoughtful examination. The causal chain between the scientific events of the late 1990s and early 2000s (Dolly’s cloning, the Human Genome Project) and the narrative construction of the Productive Society constitutes precisely the kind of anticipatory engagement that ELSI’s research seeks to analyze, but which cannot be recovered by text alone without the author’s testimony (Londoño-Proaño, 2026b).

Second, the position of an author from the Global South analyzing his own speculative imagination about genetic futures constitutes a form of situated knowledge (Haraway, 1988) that is epistemic valuable in its own right: it recognizes the constitutive social and historical location of the knowledge produced, rather than claiming a neutral analytical position inaccessible to any real researcher.

The self-reflective component is limited to The Unproductive and is explicitly marked throughout the analysis. The other two texts are analyzed through comparative narrative analysis, without access to the authorial intention.

Methodologically, the author’s reflective voice in this article does not operate in isolation, but in dialogue with an external critical tradition established about the work. Rodrigo-Mendizábal (2014) offers an independent reading that validates the ELSI core of *The Unproductive* from the outside, allowing self-reflexive analysis to focus on dimensions inaccessible to external critique: specifically, the causal chain between the biotechnological events of the late 90s and the narrative decisions that shaped the novel.

### 3.3 Scope and limitations

This study is conceptual and qualitative. It does not employ quantitative bibliometric methods, reader reception studies, or empirical policy analysis. The three texts were selected based on: (1) their direct commitment to the technological axes of the Research Topic; (2) its representativeness of the neo-indigenist Andean tradition of science fiction documented by Prado Sejas (2018, 2023); and (3) the availability of the full texts for systematic analysis. The sample is not exhaustive of Latin American science fiction; the framework proposed in the Discussion is intended as a generative hypothesis for future research rather than as a definitive empirical statement.

## 4 Analysis

### 4.1 The unproductive: anticipatory governance of genetic stratification

#### 4.1.1 The productive society as eugenic architecture

The Unproductive builds a dystopian society in which a global corporate order, the Productive Society, has replaced democratic governance. The protagonist, Operator 220, is completely oblivious to the fact that the social architecture he inhabits is based on human cloning and genetic manipulation. Maker Robert Zach articulates the fundamental logic of order in terms that unmistakably resonate with contemporary eugenics discourse: “Men and women had deviated from their true nature: work. They dedicated themselves to corrupting the species, instead of perfecting it. Let us think of a world free of human defects, which produces for everyone in an environment of peace and harmony, managing time properly. This is not a utopia” (Londoño-Proaño, 2014, p. 27).

The novel makes this manufactured origin literal. Operator 220 recalls his own “birth”:

He remembered the day he had been taken out of his ovular matrix. He opened his eyelids and the light of the room hurt his eyes. He realized he had been placed on a metal bed. […] He had been submerged in a saline aquatic medium for twenty-one years, receiving the genetic information necessary for his work as a Stock Market Operator through an umbilical cord of digital fiber (Londoño-Proaño, 2025b, p. 67).

The Operator is not born but manufactured, and his occupational function is written into him before he first opens his eyes.

As Rodrigo-Mendizábal (2014) observes in his critical analysis, the cloning industry in the novel functions as the corollary of a process of elimination of defective genes and individuals, the utopia of a fascist machine that absorbs bodies into a perfect political apparatus. This reading, carried out independently of the author, confirms that the novel frames, from a Latin American standpoint, the ELSI concern that contemporary debates on CRISPR and polygenic screening have made urgent: the resurgence of eugenic logics under the pretext of biomedical and corporate optimization (Parens and Asch, 2000).

The comparison with Huxley’s (1932) Brave New World is unavoidable, and Endara (2025) draws it explicitly. Both novels imagine the industrial production of human beings, populations of genetically identical individuals, and a pharmacological instrument of consent: Huxley’s soma finds its counterpart in Boxín. The two societies differ, however, in where they locate stratification. In Huxley’s World State, social hierarchy is fixed before birth: the Hatchery assigns each embryo to a caste, from Alpha to Epsilon, and conditions it to accept that place, so that stability rests on the absence of mobility. The Productive Society proclaims the opposite. As Operator 220 recites to himself, “there are no differences of any kind, neither racial, nor economic, nor political, nor ethnic, we are all equal. […] If you are productive you can become a true Maker. We all compete under the same conditions” (Londoño-Proaño, 2025b, p. 50). Hierarchy is not assigned at birth but produced afterwards, through permanent competition among identical clones, and enforced by a genetic surveillance that converts underperformance into a defect and the defective into disposable bodies. Huxley’s dystopia is a society of fixed castes that functions; Londoño-Proaño’s is a society of formal equality that discards those who fall behind, and that, as the sterility crisis shows, ultimately consumes itself. The ELSI concern therefore shifts from genetic predestination to genetic meritocracy: a regime in which the promise of equal opportunity becomes the mechanism of exclusion.

Endara (2025) expands on this reading by connecting the cloning system described in the novel with contemporary AI-driven homogenization: just as the cloning of identical productive bodies reduces human possibility to a single functional template, AI-generated content is producing a homogenization and emptying of meaning in language and image. The novel anticipates the dilemmas of genetic stratification, and the ELSI’s broader concern with technological, biological, or digital systems, that optimize by eliminating variation and, in doing so, eliminate the constitutive imperfection of the human being.

The parallel with Anglo-American science fiction is interesting. Greenbaum (2022) discusses Black Mirror’s USS Callister, an episode in which software clones of real individuals, created from stolen DNA, are trapped as slaves within a programmer’s virtual world, as a teachable ELSI case study on DNA ownership, the intellectual personality of AI, and the ethics of biological copying. The Unproductive constructs a similar scenario from a different standpoint: the clones are not trapped in a virtual world, but in a material corporate social order, and their slavery is not presented as a technological aberration, but as the logical end point of globalized capitalism, as mentioned by Rodrigo-Mendizábal (2014). The structural difference between the two narratives encodes a structural difference from the ELSI perspective. Whereas USS Callister frames cloning as an individual ethical violation, *The Unproductive* presents it as a systemic social condition, precisely the kind of analytical distinction that a pluriversal ELSI framework needs.

#### 4.1.2 Cloning, DNA surveillance, and the disposable body

The technological structure of the Productive Society is based on three interconnected systems: genetic manipulation for labor optimization, human cloning for labor reproduction, and DNA-based surveillance for social control. In the novel, genetic analysis is the primary instrument of criminal investigation. When a young Operator disappears, the Detectors may declare a “code orange,” the collection of DNA samples from every worker on the floor, and a news broadcast later reports the resolution of a fraud case in exactly these terms:

In their investigation, the Detectors declared floor 95 under code orange. They took DNA samples from all the Operators and analyzed them. Today they closed the case. Through their analyses they detected a strange anomaly in Operator 410, which caused him to commit fraud in his transactions. They arrested the defective employee and sent him to the Institute of Genetics. At the Institute of Genetics they carried out several studies and discovered that Operator 410 had suffered a progressive mutation of the A27 gene. It should be noted that this gene carries the information for honesty, loyalty, and respect (Londoño-Proaño, 2025b, pp. 43–44).

The fraud is not investigated as an act but diagnosed as a genotype: the crime is explained by a gene, and the offender is described as a “defective employee.” As Rodrigo-Mendizábal (2014) summarizes, the police “go directly to the genetic code to see the anomalies and from there criminalize.” More broadly, the system does not wait for a crime to occur, but uses the individual’s own genetic code as advance evidence of his or her propensity to deviate from the productive norm.

This narrative infrastructure resonates closely with three ELSI debates that are now central to the governance of genetic data. First, current predictive policing algorithms already use forensic DNA databases to generate risk profiles and target surveillance toward specific populations, a use that has been documented as a form of structural discrimination that disproportionately affects communities already overrepresented in criminal justice systems (Rothstein, 2008; Murphy, 2018). Second, polygenic risk scores, which estimate the likelihood that a person will develop certain conditions or traits based on their genetic profile, have generated concern among bioethicists precisely because they turn a statistical probability into a criterion of social classification, with applications that extend beyond the original medical context for which they were designed (Lewis and Vassos, 2020). Third, the use of genomic data by insurers and employers to assess an individual’s risk has been subject to specific regulation in several countries such as the Genetic Information Nondiscrimination Act (GINA) in the United States, precisely because it poses the risk that a person’s social and economic value is determined by their genetic makeup rather than by their actions (Rothstein, 2008).

In this sense, the novel not only imagines futuristic genetic surveillance, but anticipates a mechanism that ELSI literature has identified as central to genetic discrimination: the substitution of judgment about behaviors for judgment about biological constitutions, which shifts legal and social responsibility from what people do to what people are genetically (Parens and Asch, 2000).

The novel is explicit about what happens to bodies classified as unproductive. When the Detectors question Operator 220, by then a floor manager, about the emotion he felt when congratulating a female Operator, he weighs whether to confess:

If I confess, surely they will accuse me of being Unproductive, he said to himself. They will arrest me and sentence me to either of the two places reserved for the Unproductive. The Unproductive who are taken to the Organ Bank undergo examinations of their vital organs to verify their condition, and are then operated on to extract the organs that are in perfect condition and can be transplanted into a Building Manager or a Zone Manager. Once the organs are removed, they are injected so that they may have “eternal rest.” The Unproductive taken to the Institute of Genetics, by contrast, are considered “guinea pigs” for new genetic discoveries: for example, they are injected with lethal viruses and genetic tests are then carried out in search of the cure. Most of them die (Londoño-Proaño, 2025b, pp. 62–63).

The unproductive body is recycled as organ supply for the managerial class and as experimental material for the genetic institutions, and in Operator 220’s case the trigger for this classification is an emotion.

The cloning system reveals a deeper concern of the ELSI regarding genetic homogenization. In the novel, a geneticist discovers that high doses of Boxín, the drug the Productive Society distributes to raise productivity (discussed below), have produced two irreversible mutations, which his analyses then find repeated across the entire population (Londoño-Proaño, 2025b, p. 75): one genetic alteration affects behavior, while a second, more transcendental one causes irreversible sterility in 99.9% of the population, leaving only 0.1% capable of producing viable sperm and eggs for the periodic genetic renewal of the species (Londoño-Proaño, 2014). To aggravate this crisis, the novel establishes that clones do not begin life at zero years, but inherit the accumulated biological age of their donor lineage (Londoño-Proaño, 2014). The life expectancy of each successive clone starts out progressively longer than the previous one, causing accelerated cell degeneration, physical mutations, and decreased cognitive ability over generations (Londoño-Proaño, 2014).

The report of the geneticist, Doctor Summers, reads:

I continued my research and reached two conclusive findings. First, the mutation of the N35 gene produced the alteration in the behavior of Unproductive 410; and second, the mutation of the A27 gene produced irreversible sterility. At that moment I thought it might be an isolated case. So I collected samples from several groups. I then ran the analyses and found that these alterations were repeated in all of the samples. I made the relevant calculations, tabulated the information, and deduced that 99.9% of the population suffers from irreversible sterility. I report that the next delivery of sperm and ova, which normally takes place every five decades and allows us to rejuvenate the species, cannot be carried out unless that 0.1% is found. Otherwise, we will gradually become extinct. […] Some years ago our Institute demonstrated that the life of clones begins at the age of the donor. For example, any Operator has a useful life of forty years; if he is cloned, the life of this first clone starts at forty-five. The clone of this one will start at ninety; the third, at one hundred and thirty-five; and the fourth, at one hundred and eighty. […] Consequently, there would be an enormous mass of Unproductive Operators, and the system would collapse (Londoño-Proaño, 2025b, pp. 76–77).

The novel’s treatment of mass infertility can be read against two well-known speculative works that share the premise. In Atwood’s (1985) The Handmaid’s Tale, widespread infertility is attributed to environmental contamination: toxic chemicals, radiation, and disease. The theocratic regime of Gilead responds by appropriating the bodies of the remaining fertile women as reproductive resources. In James’s (1992) The Children of Men, adapted for film by Cuarón (2006), global infertility has no identifiable cause, and its unexplained character turns humanity’s extinction into a metaphysical and political crisis rather than a technical one. The Unproductive differs from both in the origin of the catastrophe. Sterility is neither an environmental accident nor a mystery: the social order produces it through its own logic, specifically through the drug distributed to raise productivity and the serial cloning that sustains the workforce. In the first two works, infertility befalls societies from outside or from nowhere. In the novel, it is the direct cost of an optimization regime. The governance response nevertheless converges with Gilead’s: Summers closes his report by recommending “an analysis of the entire population to discover the fertile persons” (Londoño-Proaño, 2025b, p. 77), anticipating a regime in which fertile bodies become a scarce resource to be located and administered. From an ELSI perspective, the contrast moves the question from how a society should respond to reproductive catastrophe to who is accountable when the catastrophe is engineered (Diakopoulos, 2016) by the system meant to govern it.

Together, these two mechanisms—near-total sterility and generational biological decay—mean that the Productive Society’s pursuit of genetically optimized and maximally efficient bodies is structurally self-destructive. The same optimization that ensures short-term productivity destroys the long-term reproductive viability of the population. This narrative view, that the genetic homogenization pursued for productivity carries an inherent risk of reproductive collapse, anticipates scientific concerns about genomic diversity and the evolutionary value of genetic variation (Lander et al., 2019).

The narrative mechanism of genetic homogenization sought for productivity that results in reproductive collapse reflects an established concern in population genetics. Reducing genetic diversity limits a population’s ability to adapt to environmental change, disease, and other selective pressures, because adaptive potential depends on the availability of varied genetic material in a population rather than on the optimization of a particular genotype (Frankham, 2005). A population selected or modified for a reduced set of “optimal” traits becomes genetically uniform and consequently more vulnerable to catastrophic failure at the population level, the same underlying dynamics that make monocultures vulnerable to disease outbreaks that a genetically diverse population would survive (Frankham et al., 2002). The fictional mechanism of the novel, in which a genetic mutation optimized for productive uniformity produces near-total sterility, functions as a narrative dramatization of this principle. Uniformity bought at the expense of diversity increases systemic fragility rather than resilience.

This concern has direct contemporary relevance to human germline editing. Lander et al. (2019), in their widely cited call for a moratorium on hereditary human genome editing, warn that modifying the human germline on a large scale carries risks precisely because the long-term population-level consequences of reducing genetic variation in the human gene pool are not completely predictable or reversible, an irreversibility that the novel anticipates through its own fictitious mechanism of engineered sterility. Whereas debates about governance in the real world focus on the uncertainty and irreversibility of germinal intervention, *The Unproductive* dramatizes a worse realization of that uncertainty: a society that optimized human genetics for productivity only to discover, too late, that it had brought about its own reproductive extinction.

Crucially, from a Global South perspective, the novel’s most distinctive ELSI contribution is its insistence that the populations most vulnerable to genetic stratification are not hypothetical humans of the future, but historically existing communities already designated as unproductive by the economic systems that preceded genetic governance.

The critical reception of the novel anticipates this systemic dimension. Rodrigo-Mendizábal (2014) points out that the systemic failure of the cloning model, the genetic degeneration that reduces the chances of human life to 0.1%, illustrates what Heidegger called the “concealment of danger” inherent in modern technology: continuous genetic manipulation to produce identical bodies reveals its own risk of annihilating future lives. This external reading reinforces the self-reflexive argument: the novel anticipated the dilemmas of corporate eugenics, and its fundamental internal contradiction.

Endara (2025) identifies the novel’s system of pharmaceutical control as a mechanism of psychological and political domination that operates through the consent of the population rather than through overt coercion. In the novel, the Productive Society freely distributes a genetically modified drug called Boxín, marketed as the “Nectar of Production”, described as favored by cell regeneration and increased consumer productivity (Londoño-Proaño, 2014). Pharmacologically, Boxín acts on the nervous system to induce a state that the characters in the novel call virtual happiness. The mechanism of domination works through three intertwined functions. First, consumption is legally unrestricted and socially normalized. Almost all Operators consume Boxín willingly, and one character points out with silent pride that he has remained free of that substance, marking abstention, not use, as a deviant condition. Second, the drug is economically integrated into the financial system of the Productive Society. Its manufacturing laboratories are listed on the stock exchange and the Operators speculate on their shares, making the population’s own economic interest complicit in the continuous distribution of the drug. Third, the security system, the Detectors that monitor and interrogate the population, treats the use of Boxín with a structured leniency. A single scene places the three features side by side. As Operator 220 watches a colleague being called in for questioning, he imagines the encounter and then turns to the stock market:

He thought it would not be at all strange for that stock-market employee to declare himself guilty of having ingested unauthorized quantities of Boxín. The Detectors would show a compassionate smile and tell him not to worry, that it happened frequently, that he had committed no crime. “As you surely know, Operator 349,” the Detectors would tell him, “Boxín is a substance of free consumption.” The Operator would breathe a sigh of relief and apologize for the impertinence. On his holographic screen an icon flickered, indicating that a block of shares had been put up for sale on the virtual market. He checked its origin. It was a package offered by an Operator from the West zone: barely ten shares in one of the laboratories that produce Boxín. It seemed to him that his prayers had been answered. Those laboratories gain value every day, he told himself; they make a mass-consumption genetic product. The Global Corporation itself distributes it free of charge, in doses of twenty ampoules a month… (Londoño-Proaño, 2025b, pp. 32–33).

The Detectors’ leniency, the free distribution of the drug by the Global Corporation, and the Operators’ financial stake in its production appear in a single sequence: the protagonist’s first reaction to a colleague’s interrogation is to speculate on Boxín shares.

Together, these characteristics constitute what Endara (2025) interprets as a system of domination considerably more effective than direct repression. Instead of forcibly suppressing the critical faculty of the population, the Productive Society induces its citizens to voluntarily and continuously anesthetize themselves to that faculty, in exchange for high productivity and pharmacologically induced satisfaction. This narrative mechanism anticipates contemporary ELSI debates about neurotechnology and cognitive enhancement, in which the central question of governance is not just whether pharmacological or neural enhancement is technically possible, but whose interests are served when a population’s capacity for critical dissent is chemically diminished under the voluntary guise of well-being and productivity (Farah, 2002).

The ELSI implications of biological copying go beyond genetic stratification, entering the realm of consciousness and personal identity. Freund et al. (2023) identify copying consciousness as a “probable and imminent” ELSI challenge, one that raises questions about the identity, moral status, and governance of entities whose subjective experience may have been duplicated or artificially generated. The Unproductive anticipates precisely this problem: the clones of the Productive Society are not simply biological copies, but entities whose capacity for consciousness, memory and autonomous identity is systematically suppressed in the service of productive homogeneity. The novel thus frames this ELSI concern from the side of the copied rather than the copier: copying biological substrates without governance frameworks for the resulting conscious entities produces not people, but instruments.

I can speak directly to the narrative choices that shaped this novel, as I am both its author and, in this article, its analyst. From 2001 onwards, my involvement with human cloning and the Human Genome Project became an obsession. I spent nights trying to understand the mechanism behind Dolly’s cloning (Londoño-Proaño, 2026a). Somatic cell nuclear transfer, the technique of emptying an egg and filling it with the genetic instructions of another being. That empty egg did not seem like a lab procedure to me; it read like a blank canvas for market ambition. If science could already reprogram cells to design living beings according to specifications, I wondered what would prevent corporations from doing the same with human beings, optimizing us to maximize profitability (Londoño-Proaño, 2026a). The presentation of the map of the human genome in those same years deepened this concern instead of resolving it. Seeing the human essence reduced to a sequence of four chemical letters made me understand that an emerging economic logic would not be satisfied with owning only our labor time, but would seek to possess our own biological code.

This is the specific reason behind several of the novel’s central design decisions. I deliberately rejected the imagery of conventional mechanical robots in the face of dystopian science fiction in favor of genetic manipulation and engineered babies, because I wanted the technology to be invisible, internal, and inherited, rather than externally and visibly mechanical, a threat encoded in one’s own body and not imposed from the outside. I envisioned human factories in which any defect in a baby’s DNA, undetectable to the naked eye, would doom that child to be discarded as a defective byproduct. This image became the narrative seed of the novel’s system of genetic stratification and its population of unproductive.

This image invites comparison with Niccol’s (1997) Gattaca, in which genetically engineered “valids” monopolize opportunity while naturally conceived “in-valids” are relegated to menial work, and genetic discrimination, though formally illegal, is routinely practiced through DNA testing. In Gattaca, however, the excluded stand outside the enhancement system, and the protagonist can still evade it by borrowing another man’s genetic identity. In *The Unproductive*, there is no outside: every body is manufactured, and the defect that condemns it is produced within the system itself.

I felt an urgency to dramatize a future in which the right to live depended on passing a corporate quality control inspection, a deliberate reversal in which biological birth and industrial manufacturing become indistinguishable. The decision to extend this logic specifically to cloned populations, rather than to genetically modified but still individually born humans, stemmed directly from this concern. Cloning allowed me to dramatize a society in which human reproduction itself had been absorbed into corporate production cycles, collapsing the distinction between making a product and creating a person (Londoño-Proaño, 2026a).

### 4.2 Quipucamayoc: anticipatory governance of algorithmic systems

#### 4.2.1 The quipu as proto-computational system

*Quipucamayoc* follows Pomacha, the young son of Maillama, the defeated chief (*curacá*) of the Guacro, a coastal town conquered by the Inca empire. After witnessing the subjugation of his father, the twelve-year-old Pomacha vows revenge on the Inca and, with calculated patience, becomes an apprentice to Kuntur Ñahui, the Inca official, a *quipucamayoc*, sent to catalog the wealth taken from his people. Kuntur Ñahui, initially resentful of his fate on the coast, comes to regard Pomacha as a surrogate son and, against his own judgment, teaches him the whole art of quipu. Not just the recording of censuses, harvests, and military strength, but the much more sensitive knowledge of how quipus could be selectively falsified, corrected, or concealed, secrets which, the story specifies, were unknown even to the Inca’s priests or to the Inca himself.

The story renders this transmission of restricted knowledge as a lesson in Kuntur Ñahui’s voice:

“Put those two quipus together. Like that, very good. Now tie the last fringe, but with the knot facing you. Do you notice anything? Nothing, good. Now do the same, but with the knot turned the other way. Ah, a sign! What does it tell you? Two lines without a knot? What could that be for? Do what the knots tell you… Ha, ha, ha! Well, there you have how many children the previous Inca really had. Do they seem like a lot to you? Look closely at the quipu, there is an empty space there… any knot will tell you that in just one year, almost all of those children died. Ha, ha, ha! Those are things better not spoken of, young apprentice…” At times Pomacha could not believe how many things the quipus could say. With pride, he soon discovered that he knew more about the true history of the Incas than the self-proclaimed children of the Sun themselves (Salvo, 2014, p. 165).

The quipu holds a double record: an official surface, and hidden layers readable only by those who know the encoding conventions. It is a data system whose full content is inaccessible to the political authority it serves.

The technical core of the story is what the characters call the “bad knot.” A knot that bears a hidden signature that forces any quipucamayoc who finds it to replicate it in each subsequent quipu they produce. Because the quipu system allowed for limited chains of modification, one quipu could be corrected by another quipucamayoc without erasing the original maker’s mark, but only up to five times, the bad knot exploited this same controlled editing protocol, spreading silently from cord to cord, quipu to quipu, region to region, over an unspecified period of years, until falsified data could no longer be distinguished from reliable data. In the story’s own diagnostic terms, a quipu with enough bad knots ceases to encode true information and becomes a simple set of ropes without any meaning (Salvo, 2014).

Yucraj, the quipucamayoc who later diagnoses the crisis, explains the mechanism to his lord:

“What a quipu says can be interpreted by any quipucamayoc, who in turn can modify what is in the quipu, according to how things have happened. He can correct. He can change.” […] “A quipucamayoc places his mark on every quipu he makes. A quipu belongs to its quipucamayoc, and if it is modified by another quipucamayoc, the modification does not alter the mark of origin, although this holds only for five modifications. […] My lord, some very wise quipucamayoc wove the bad knots somewhere, and who knows how long ago. Knots that he put in one quipu were repeated in another, then in another, and met again… […] The bad knots carry a sign that tells the quipucamayocs they must repeat them in every quipu they make. They repeat and repeat, it seems for years… And there is no way to correct them. The bad knot untied from one quipu has already been tied into another” (Salvo, 2014, p. 169–171).

The explanation specifies the three properties that make the sabotage undetectable and irreversible: it exploits a legitimate editing protocol, it carries an instruction for its own replication, and it propagates faster than it can be removed.

Kuntur Ñahui is finally executed for a betrayal he did not commit, victim of court intrigues and rival factions that exploited his closeness to Pomacha, and the Inca, in an act of calculated clemency, spares Pomacha’s life while permanently confining him to his coastal palace as the official quipucamayoc of the region, installing him as Guacro Capac. The same colonial administration position that his people were forced to accept. Only after this appointment does Pomacha, now embittered by the death of Kuntur Ñahui and freed from any remaining allegiance to the empire, deliberately begin to sow the bad knots in the imperial information system, using, with calculated irony, the very knowledge that the imperial official himself had entrusted to him. Sabotage, therefore, is neither accidental nor anonymous in its origin. It is a slow, intergenerational, and deliberate act of information warfare, executed by a colonized subject who inherited privileged access to the colonizer’s central data infrastructure and used that access to corrupt it from within, for a period of time too long for a single administrator to detect its origin.

By the time the narrative reaches Yucraj, a quipucamayoc who serves another regional lord one or more generations later, the corruption has become total. “No one knew the origin of the bad knot, only its disastrous consequences” (Salvo, 2014, p. 169). The consequence for governance is precisely stated. The empire has lost access to its own military intelligence on conquered kingdoms, its harvest records, and its vital statistics on births and deaths. In short, all the administrative memory that had sustained the Inca imperial control. Yucraj’s lord immediately understands the importance, if not the technical mechanism. The empire, in his words, is now a snail without its shell (Salvo, 2014).

“My lord, there is nothing left to do. All the quipus of the empire that have been used to carry reports from one place to another are useless now. The empire has lost its tongue, its words, its thoughts. We would have to burn all the quipus and start new ones…” […] He had not understood much of what the quipucamayoc had told him, but one thing was clear: the Inca empire lacked all the information that had kept it running until that moment. The records of the military strength of the conquered kingdoms no longer existed; there was no knowledge of recent harvests; it was impossible to know how many people had died and how many had been born in recent years. In short, one of the principal instruments of Inca power, which had served efficiently for years to maintain the dominance of the children of the Sun over other peoples, no longer existed (Salvo, 2014, p. 171–172).

Yucraj takes advantage of this vulnerability to incite his lord’s rebellion, and the resulting civil war between the brothers Atahualpa and Huascar. The true historically documented succession crisis of the empire explodes in the story just at the moment when the information infrastructure collapses, just before the arrival of the *viracochas*, the Spanish conquistadors. In the final scene of the story, the dying Guacro Capac, Pomacha, decades later, learns with quiet satisfaction of Atahualpa’s victory over Huascar, reflecting that the collapse of the empire is entirely due to the bad knots of Kuntur Ñahui.

In the palace of Cerro Azul, an aged and dying Guacro Cápac contemplated the sunset on the shore of the sea. With poorly concealed joy, he received the news of the successful rebellion that had ended with the capture of Huáscar and the enthronement of his brother Atahualpa as the new Inca. The power of the children of the Sun was cracking. […] And to think that he owed it all to Kuntur Ñahui and his bad knots, something that should never have been done… (Salvo, 2014, p.172–173).

This narrative structure, a single act of data corruption, introduced by an insider with legitimate access to the system, which silently and irreversibly spreads through the entire information infrastructure of an empire for years or decades, until the capacity for governance collapses at the precise historical moment of maximum external vulnerability, It constitutes a close narrative counterpart to of what contemporary cybersecurity research identifies as an advanced and persistent threat. A slow, low-visibility, insider-enabled compromise designed to remain undetectable until its cumulative effect is irreversible (Brundage et al., 2018). The story also specifies the exact damage to governance that such an attack produces. Not the loss of a single decision, but the loss of the underlying data infrastructure that makes any future decision possible.

#### 4.2.2 The bad knot: systemic algorithmic failure and epistemic sovereignty

The central crisis of the story, a “bad knot” that spreads virally through the empire’s entire quipu network, corrupting its data irreversibly, constitutes a narrative model of systemic algorithmic failure. Its properties mirror those identified in contemporary research on cybersecurity and AI governance: self-replicating, obscuring the origin, with systemic-level effects that are impossible to reverse once established (Brundage et al., 2018). The political consequence is total: as Yucraj puts it in the passage quoted above, the empire has lost its tongue, its words, its thoughts.

A secondary but significant dimension of the Quipucamayoc ELSI concerns the deliberate destruction of a rival information system. In a brief but incisive passage, Kuntur Ñahui recalls the extermination of a people who knew how to engrave signs on lima beans (*pallares*), and approves of it:

The quipucamayocs recorded the information that fed the Tawantinsuyo. Thanks to the quipus, the Incas had achieved their present supremacy over other peoples, for they made it possible to store information reaching back to periods that the common people considered mythical. Wars, droughts, famines, journeys, storehouses, crops: all this information could be stored in a quipu and deciphered by any quipucamayoc. It was not knowledge accessible to everyone. It should not be. Kuntur Ñahui recalled, as an example, the quipu that recorded the atrocious death of the people of other nations who knew how to engrave signs on lima beans, signs that said things, not like the huacas or the quipus, but with greater certainty and clarity. He judged wise the Inca who had ordered the elimination of those who could preserve a perennial truth different from the truth recorded by the Incas (Salvo, 2014, p. 160–161).

This specific passage is the exact mechanism of what De Sousa Santos (2010) calls epistemicide. Not simply the abandonment or gradual displacement of a rival knowledge system, but its violent and deliberate elimination by a dominant power precisely because that system offered a documentary standard, described in the text as greater certainty and clarity, capable of questioning the official record. The passage makes it clear that the threat posed by the peoples of the engraving was not military or economic, but epistemic. They possessed an alternative technology of truth-preservation outside of imperial control, and the Inca state found it necessary to destroy those who possessed that knowledge rather than risk a competing record of events that would survive alongside their own. Kuntur Ñahui’s approving judgment on this extermination, pronounced in the same sentence in which he reflects on his own privileged and restricted access to the knowledge of the quipu, reveals a governing logic in which the monopoly of information is treated as inseparable from political control. A rival infrastructure of truth is uncomfortable for the empire, and constitutes an existential threat to its authority, justifying the same violent response as an armed insurrection.

This detail connects the story to ELSI’s contemporary debates on the governance of knowledge infrastructures with unusual specificity. While the “bad knot” narrative (Section 4.2.2) dramatizes the corruption of an information system from within, this second episode dramatizes the deliberate destruction of an alternative system from the outside, together offering two distinct but complementary models of how governing powers can lose or eliminate access to reliable knowledge. The extermination of the bean engravers raises questions directly relevant to current debates about the governance of AI training data, the underrepresentation of non-Western knowledge systems and languages in large language models, and the digital preservation of indigenous epistemic traditions. In each case, the question that the story raises, who has the authority to determine what systems of knowledge may exist, remains structurally unresolved.

### 4.3 El Yachak: anticipatory governance of biotechnology and human enhancement

#### 4.3.1 Embodied biotechnology as alternative enhancement framework

*The Yachak* narrates the healing of a starship in crisis by José Sánchez, an elderly Kichwa yachak from the Mojanda highlands, near the sacred Peguche waterfall. The story begins with José Sánchez perceiving, through his own body, that *Pachamama* (Mother Earth) is sick. The mountaintop feels warm when it should be cold, the ravines exhale a warm, sickly wind instead of their usual cold, and the guinea pigs in their hut remain mesmerized and motionless, reflecting the disturbance. In a parallel narrative thread, an alien ship manned by Orkyyun’s TSKZZ, shapeshifting beings whose consciousness migrates between eight tubular limbs and a bulbous central body, and whose perfectly programmed and controlled society is governed by pseudo-artificial minds, has been taken over by an uncontrollable affliction that spreads throughout the ship and that the narrator simply calls fear, which causes crew and ship to go out of control to unexplored regions of the galaxy. The ship, now abandoned and dying, drifts toward Earth and the three sacred peaks that anchor the Yachak’s cosmology: Cayambe, Cotacachi and Imbabura.

The story’s description of the TSKZZ civilization is significant for an ELSI reading:

The tiny TSKZZ of Orkyyun, passengers of the frightened living ship, were beings in continuous mutation. […] Their society was one of the most exact and programmed in the Universe. They were expert builders of machines which, endowed with the characteristics of life, mixed cells, molecules, electronic units, and subatomic fluids. They handed control of their existence over to their creations, whose pseudo-artificial minds governed their society, their trade voyages, their treaties of techno-economic exchange with other civilizations of the galaxy, and their telepathic-genetic reproduction. In the universe of the TSKZZ everything was controlled, timed, measured. Everything, until FEAR took hold of them. […] When FEAR attacked them, nothing could save them (Páez, 2007).

The civilization that collapses is the one that has delegated governance, commerce, and even reproduction to pseudo-artificial minds: a society of total optimization with no internal resource for the affliction that strikes it. The cure, as the story goes on to show, has to come from outside that paradigm.

Diagnosing the disorder requires a specific and detailed ritual practice, which the story describes in careful ethnographic detail. The Yachak arranges a ceremonial mat of sacred stones, obsidian, quartz and river stones, placed at precise distances from each other to reproduce the world and the forces that constitute it, together with a deliberately syncretic arrangement of objects. A Christian crucifix and images of saints placed next to a pre-Columbian obsidian arrowhead, ceremonial spears, an ancient bayonet from the wars of independence, and a rosary, invoking both Christian deities and the ancestral gods of their people. Accompanied by his son and apprentice Lluntu, the Yachak spends the night battling winds and malevolent spirits, using lit candles and mouthfuls of liquor (*aguardiente*) blown through the candle flame to repel them, and eventually sends Lluntu on a dangerous mission to the Quebrada Negra (Black Ravine) to retrieve a small stone that glows blue, green, and red, the physical manifestation, within the ritual framework of the Yachak, of the damaged alien ship itself, which the narrative presents as no greater than the largest of its ceremonial stones.

The story describes the altar in these terms:

On a white cloth, the Yachak had laid out his stones in their exact positions. Each stood at the proper distance from the others; together they reproduced the world and the forces that constitute it. […] In the center of the cloth gleamed, jet-black, an obsidian arrowhead, perfect, sharp, prehistoric. These were the stones of power. Among them the Yachak had placed various objects: pieces of iron, copper, and wood, a transparent piece of crystal that reflected the flame of two candles set to the right and left, images of saints, a marble crucifix, photographs of himself in the company of priests or doctors. Facing the Shaman, on the opposite side of the cloth, an old and rusted bayonet from the wars of independence was driven into the ground, along with two chonta-wood spears adorned with multicolored feathers […]. All the energies of the world were reproduced and concentrated on the Yachak’s table; that is why he could heal, know, control… (Páez, 2007).

The altar incorporates, rather than excludes, the institutions of the Church and of Western medicine, including photographs of the Yachak with doctors: his system is not pre-modern but syncretic, absorbing biomedicine as one more force among others.

Once the stone vessel is brought before him, the Yachak communicates directly with his pilot, mind to mind. Crucially, the story situates this not as a scientific diagnosis, but as a traditional Andean ethnomedical diagnosis. The Yachak asks the alien what affects him and, upon hearing his answer, recognizes the condition precisely as *espanto* (soul-fright), a culturally specific Andean folk disease understood as a catastrophic loss of harmony, specifically the breakdown of the balance that normally keeps heat and cold in separate balance within a living system. After identifying the ailment, the Yachak performs a formal invocation of healing, invoking by name the surrounding sacred mountains and a syncretic pantheon of Catholic saints and Andean guardian spirits, while continuing to blow liquor over the vessel. The narrative then describes the cure in terms that deliberately fuse ethnomedical and technological registers:

The corrosive rain cleansed the ship’s bioelectronic channels like a balm. The energy found its domains and flowed through the proper subatomic circuits. An infinite stillness took hold of the extraterrestrial and his companions. The Yachak’s word, like a soothing massage, put the computers’ contacts in order and restored the molecular-electronic management of the data (Páez, 2007).

The ship recovers, rises and leaves, its lights briefly illuminating the waterfall before disappearing into the night.

As Londoño-Proaño (2025a) has argued, this healing mechanism constitutes a form of organic biotechnology. Instead of repairing the ship through instrumental technical intervention, diagnostic scanning, mechanical repair or designed countermeasures, the Yachak restores its function through a ritual protocol whose therapeutic logic is relational and harmonic rather than mechanical, treating the ship’s bioelectronic failure as something curable with the same ethnomedical framework used to treat human and terrestrial ailments.

From ELSI’s perspective, the story proposes an alternative framework for enhancement that deviates fundamentally from the dominant transhumanist paradigm in the bioethical debate. While transhumanism proposes the expansion of individual capacities beyond the typical norms of the species (Bostrom and Sandberg, 2009), the yachak model proposes the restoration of systemic harmony between the individual body, the social body, the natural environment, and the cosmos. This distinction has direct implications for governance. It raises questions about what is considered valid biotechnological knowledge, who is authorized to intervene in biological systems, and what the relationship between individual well-being and ecosystem health should be in governance frameworks.

#### 4.3.2 Contact as collaboration: reframing human-non-human intelligence relations

El Yachak’s most distinctive contribution to ELSI discourse lies in its model of contact between human and non-human intelligence. In the dominant tradition of Western science fiction, and in the ELSI literature on artificial intelligence and extraterrestrial life, contact scenarios are often framed around power asymmetry. The alien is a potential threat to be defended against, or a resource to be exploited or negotiated, and governance frameworks are accordingly designed to protect human interests.

*The Yachak* proposes a structurally different model, specific to the crew and craft depicted in the story, rather than to extraterrestrial life in general. The crew of the TSKZZ of this single damaged ship are not agents of conquest, negotiation or threat. They are, within the ethnomedical framework of the story itself, those affected by a disease, the part that needs diagnosis and treatment, occupying the same narrative role that a sick human patient would have in a healing ritual. Consequently, the Yachak is not an advocate, negotiator, or victim of contact, but a healer whose specific ethnomedical knowledge, his ability to recognize *espanto* and restore harmonic balance through ritual invocation, is what makes the encounter survivable for both parties. The relationship that the story stages is therefore neither conquest, nor exchange, nor defense, but a single act of medical care extended to a foreign patient. That is, mutual vulnerability (the dying ship of the crew; the Yachak’s own son endangered by the same disturbance) resolved by reciprocal necessity rather than by domination or negotiated self-interest.

The exchange between the Yachak and the pilot stages this mutual vulnerability directly:

“You have made my world sick,” said the Yachak, “you have made my son sick.”

“Forgive us,” replied the pilot, almost fainting in the control room of his ship, “we are not accustomed to harming other living beings, but now we are sick and we have transmitted the sickness to your world.”

“Go away!”

“I cannot. We will die soon.”

“And your sickness will stay and finish off my world.”

“Yes.”

Both fell silent. The Yachak felt the pain of the demon in the colored stone; the pilot perceived the suffering of that world which, without meaning to, he would destroy.

“Who are you?” asked the Yachak.

“A traveler,” answered the demon. “And you?”

“A Yachak, a healer.”

“Heal me.”

José Sánchez thought about it intensely. That was the only hope for the Earth, for his son.

“What is wrong with you?”

“Fear.”

“*Espanto*?”

“Yes” (Páez, 2007).

Neither party can impose a solution: expulsion is impossible, and the illness will outlive the ship. The resolution comes through a request for care (“Heal me”) and a diagnosis made in the healer’s own nosological categories.

This approach offers a distinct standpoint on a dimension of the contemporary debate on AI alignment that is central to ELSI’s research. The possibility that non-human intelligences, whether extraterrestrial or artificial, may occupy the role of a vulnerable party requiring attention rather than an adversarial party requiring containment, and that the appropriate governance framework for such an encounter may be therapeutic rather than regulatory or defensive.

The healing capacity of the yachak is inseparable from its position within a specific relational network: its knowledge of the Mojanda mountain, the Cayambe and Cotacachi volcanoes, the Peguche waterfall and the spiritual landscape of the northern Andes. This situated knowledge (Haraway, 1988) cannot be abstracted from its ecological and cultural context, anticipating ELSI’s contemporary debates on the appropriation of indigenous biotechnological knowledge by pharmaceutical corporations and the governance frameworks needed to protect indigenous intellectual property (Mgbeoji, 2006).

## 5 Discussion

### 5.1 What the analysis reveals: a different standpoint

The comparative analysis of the three texts supports the central hypothesis of the article: Latin American speculative fiction engages ELSI’s central debates on genetic engineering, algorithmic governance, and biotechnological enhancement from structural positions not available to the Anglo-American canon. Its value lies less in whether it arrived at these questions before the Global North than in the different questions it asks about the same technologies. Each text encodes ELSI’s concerns not as abstract philosophical puzzles, but as extrapolations of historical realities and presents, the expendable body, the destroyed knowledge system, the appropriate healing tradition, which grant them a clear analytical identification on governance issues that are less visible from the positions that dominate the Anglo-American canon.

Three structural features distinguish this corpus from the Star Trek tradition of the Global North. First, it situates ELSI concerns in contexts of existing structural inequality: the genetic stratification of The Unproductive is an extrapolation of present realities, not a hypothetical future threat. Second, it proposes ethical frameworks that are relational and systemic rather than individual- and rights-based, attentive to community well-being, ecological balance, and interdependence between species. Third, it brings to the fore the question of who is authorized to define what counts as technology, knowledge, and improvement, positioning non-Western epistemic systems as autonomous traditions of knowledge with governance implications, not as pre-scientific survivals awaiting incorporation into modern science.

The difference in standpoint becomes clearest when the same technology is compared across the two traditions. In Star Trek, the ELSI danger of genetic engineering is embodied by the Augments, enhanced humans whose superiority threatens a liberal federation that responds by prohibiting enhancement; the risk comes from those made more than human, and governance is the safeguard. In The Unproductive, the risk falls on those classified as less than productive, and the governing order is itself the eugenic agent. In Western cybersecurity framings, the corruption of an information system is a threat to a legitimate order; in Quipucamayoc, it is the act of a colonized subject against an imperial monopoly of knowledge, which raises the prior question of whose information order counts as legitimate. And where Star Trek’s encounters with the unknown are mediated by Starfleet technology and a doctrine of non-interference, El Yachak resolves first contact through an indigenous healer’s knowledge and an ethic of care. In each case, the Latin American text does not simply arrive at the same ELSI question earlier or later: it asks it from the position of those on whom the technology is exercised.

### 5.2 Toward a pluriversal anticipatory governance framework

Based on these findings and on the theoretical resources of Global South epistemologies (De Sousa Santos, 2010), pluriversal design (Escobar, 2018), and science fiction studies (Rieder, 2017), this article proposes a pluriversal anticipatory governance framework structured around four principles.

First, epistemic plurality provides a resource for governance. Anticipatory governance frameworks should actively seek out and engage speculative narratives of non-Western traditions as standalone governance resources, not as exotic complements to conventional analysis, that can identify problematic dimensions invisible from within the dominant tradition. Latin American, African, South Asian, and Pacific speculative fiction constitutes unexplored archives of anticipatory governance thinking.

Second, structural inequality must be integrated into the ELSI context. ELSI analysis should routinely situate the governance of emerging technologies within the structural inequalities that will determine their differential impacts. The question is not only what are the risks of this technology, but who will those risks fall on and how do existing inequalities shape the distribution of risks and benefits?

Third, self-reflexive analysis can be a valuable ELSI method. The systematic engagement of science fiction authors, especially authors from the Global South, as thoughtful analysts of their own contributions to anticipatory governance offers ELSI’s research a distinctive methodological resource: access to the ethical concerns and ideas about governance that generated speculative narratives, not just their textual products.

Fourth, relational ethics are a fundamental principle of governance. The relational and systemic ethical frameworks proposed by non-Western speculative traditions, attentive to community well-being, ecological balance, and interdependence between multiple species, offer governance principles that complement and challenge the dominant rights-based individualistic frameworks in bioethics and ELSI studies.

### 5.3 Implications for policy design

A clarification is needed before turning to specific policy implications. The contrast this article draws between the Anglo-American canon and Latin American speculative fiction should not be read as an opposition between two homogeneous blocs. Neither the Global North nor the Global South is a unified epistemic system; both are heterogeneous admixtures of worldviews, social stratifications, and historical situations that coexist in tension and co-produce the world we inhabit (Jasanoff, 2004). The corpus analyzed here makes this evident. The Yachak’s altar places Catholic saints, an obsidian arrowhead, and photographs of the healer with physicians on the same ceremonial cloth: his knowledge is not a pre-modern alternative to Western science, but a syncretic practice that absorbs and reworks it. *Quipucamayoc* shows that epistemicide is not exclusive to European colonialism, since the Inca empire itself eliminates a rival Andean writing system. *The Unproductive* locates its eugenic order not in a foreign power but in a globalized corporate capitalism that is as present in Latin America as in the North. The standpoint these texts offer is therefore not a pure outside to Western modernity, but a set of positions within a shared and unequal world from which its technologies are experienced differently. The implications below should be read in this light: the aim is not to replace one knowledge system with another, but to make governance attentive to the plurality of positions from which emerging technologies are lived, contested, and reworked (De Sousa Santos, 2010; Escobar, 2018).

The pluriversal framework has direct implications for inclusive and culturally situated policy design in an era of increasing genetic and biotechnological capacity. First, governance processes for technologies such as CRISPR, polygenic screening, and synthetic biology should actively seek input from communities whose historical experiences of eugenic violence and genetic discrimination offer a distinct perspective on the risks of genetic stratification. The communities that appear in narratives such as The Unproductive are not hypothetical victims of the future, but current political actors whose voices belong to governance processes.

Second, epistemic governance frameworks, the systems that determine whose knowledge counts as valid evidence in regulatory processes should be designed to recognize the plurality of knowledge practices that already coexist within any society, including indigenous and traditional knowledges that have developed sophisticated understandings of biological systems under governance, often in dialogue and tension with biomedical science rather than in isolation from it. The yachak’s embodied knowledge of human-environment interaction is not only culturally interesting; it can be epistemically indispensable for governance frameworks appropriate to the complexity of living biological systems.

Third, the cultural infrastructure of anticipatory governance, science fiction, speculative design, and narrative foresight activities through which societies test their technological future, should be diversified as a matter of deliberate policy. The homogenization of speculative imagination around Anglo-American franchises reduces the space for governance problems in ways that can be costly when technologies materialize in social contexts far removed from their narrative anticipations.

This homogenization is, however, already loosening. Chinese and Korean speculative fiction has entered the global mainstream: Liu Cixin’s The Three-Body Problem (Liu, 2014) won the Hugo Award for Best Novel in 2015 and was adapted as a Netflix series (Benioff et al., 2024), while Korean productions such as Bong’s (2017) Okja, on corporate genetic engineering, and Yeon’s (2023) Jung_E, on the copying of a human mind into combat AI, circulate worldwide. Together with Greenbaum's (2025) attention to Japanese narratives such as Astro Boy, these works show that the speculative archive available to anticipatory governance is already plural. The task is to ensure that this plurality also reaches traditions, such as the Latin American and Andean ones examined here, that still circulate mainly in their own languages and outside the major cultural industries.

### 5.4 Limitations and future research

This study has several limitations that future research should address. The sample of three texts, although analytically productive, is not exhaustive of Latin American or Andean science fiction. Future work should systematically map the entire corpus of Latin American science fiction with ELSI relevance to test the pluriversal framework on a large scale. The self-reflexive component, although methodologically justified, introduces an inherent limitation in the analysis of The Unproductive: the author’s testimony about his intentions cannot be independently verified and must be treated as one analytical contribution among several, not as definitive proof of the importance of the text in governance.

Moreover, the framework proposed here focuses on narrative analysis and does not address the empirical question of whether Latin American science fiction actually shapes the governance imaginaries of its readers, policymakers, or scientists in the region. Empirical studies on reception and influence, combining text analysis with reader surveys, political discourse analysis, and citation tracking, would be necessary to test the full causal claim that this corpus has functioned as anticipatory governance, rather than merely codifying governance concerns.

The framework should extend beyond Latin America to other non-Western science fiction traditions such as African, South Asian, Pacific, and East Asian, to test whether the structural features identified here are specific to the Latin American context or constitute broader features of speculative fiction produced from positions of colonial marginalization.

## 6 Conclusion

Beyond Star Trek, there is a speculative tradition that has been testing the ethical, legal, and social implications of emerging technologies from the perspective of communities for whom those implications are not hypothetical. This article has demonstrated, through the comparative analysis of three Latin American science fiction texts that this tradition engages ELSI’s debates on genetic stratification, algorithmic sovereignty, and biotechnological enhancement from a different standpoint, rooted ininequality, colonial erasure, and the historically expendable body.

The pluriversal anticipatory governance framework proposed here offers ELSI research a way to integrate this tradition systematically, not as a complement to the analysis of the North, but as an independent resource of governance that identifies problems, proposes frameworks, and embodies ethical principles that the Anglo-American canon has tended to leave at its margins. In an era of accelerating biotechnological capability, the governance frameworks that will determine who thrives and who is designated as unproductive will be shaped, in part, by the speculative imagination we bring to the task. Broadening that imagination to include the voices of the Global South is not simply an act of academic inclusion, it is a need for governance.

## Data Availability

The original contributions presented in the study are included in the article/Supplementary material, further inquiries can be directed to the corresponding author.
