# Supplementary material for: Beyond Star Trek: Latin American science fiction and the ELSI of emerging technologies
Source: Front Sociol. 2026 Oct 29;11:1898512. doi: 10.3389/fsoc.2026.1898512 (PMC13754554; doi:10.3389/fsoc.2026.1898512)
Supplement: Supplementary file 1 [file Supplementary_file_1.DOCX]

Supplementary Material

*Beyond Star Trek: Latin American Science Fiction and the ELSI of Emerging Technologies*

Cristián Londoño-Proaño

# 1 Supplementary Data

This file provides the original Spanish text of the excerpts quoted in English translation in Section 4 of the main article. Translations in the main text are by the author. Excerpts are reproduced as printed; editorial omissions are marked [...], and minimal corrections of evident typographical errors are marked with square brackets. Page numbers refer to the editions cited in the main text. Yachak was consulted in its online version, which has no pagination.

## 1.1 Los improductivos (Londoño-Proaño, 2025b)

**Supplementary Excerpt 1.** Corresponds to Section 4.1.1 of the main text (Londoño-Proaño, 2025b, p. 67).

Recordó el día que había sido sacado de su matriz ovular. Abrió los párpados y la luz de la habitación lastimó sus ojos. Se dio cuenta que había sido colocado en una cama metálica. [...] Había estado sumergido en un medio salino acuático durante veintiún años, recibiendo la información genética necesaria para su trabajo de Operador Bursátil, a través de un cordón umbilical de fibra digital.

**Supplementary Excerpt 2.** Corresponds to Section 4.1.2 of the main text (Londoño-Proaño, 2025b, pp. 43–44).

»En su investigación, los Detectores declararon al piso 95 en código naranja. Tomaron las muestras de ADN a todos los Operadores y las analizaron.
»El día de hoy pusieron punto final al caso. A través de sus análisis detectaron una extraña anomalía en el Operador 410, que ocasionó que cometiera fraude en sus transacciones. Tomaron preso al defectuoso empleado y lo enviaron al Instituto de Genética.
»En el Instinto de Genética hicieron varios estudios y descubrieron que el Operador 410 sufrió una progresiva mutación del gen A27. Cabe señalar que, este gen tiene la información de la honestidad, lealtad y respeto.

**Supplementary Excerpt 3.** Corresponds to Section 4.1.2 of the main text (Londoño-Proaño, 2025b, pp. 62–63).

Si confieso, seguramente, me acusarán de Improductivo, se dijo. Me detendrán y me condenarán a cualquier de los dos sitios destinos a los Improductivos. A los Improductivos que les llevan al Banco de Órganos les hacen estudios de sus órganos vitales para comprobar el estado y luego les practican cirugías para extraerles los órganos que se encuentren en perfectas condiciones y puedan servir para ser transplantados a un Gerente de Edificio o a un Gerente de Zona. Una vez extraídos los órganos les inyectan para que tengan «un descanso eterno». En cambio, a los Improductivos que les llevan al Instinto de Genética les consideran «conejillos de indias» de los nuevos descubrimientos genéticos, por ejemplo, les inyectan virus letales y luego hacen pruebas genéticas en la búsqueda de la cura. La mayoría de los casos muere.

**Supplementary Excerpt 4.** Corresponds to Section 4.1.2 of the main text (Londoño-Proaño, 2025b, pp. 76–77).

—Seguí mis investigaciones y obtuve dos conclusiones contundentes —escuchó al doctor Summers—. Primero, la mutación del gen N35 produjo la alteración en el comportamiento del Improductivo 410; y segundo, la del gen A27 produjo una esterilidad irreversible.
»En ese instante pensé que quizás se trataba de un caso aislado. Entonces, recolecté muestras de varios grupos. Luego hice los análisis y supe que dichas alteraciones se repetían en la totalidad de las muestras. Realicé los cálculos pertinentes, tabulé la información y deduje que el 99,9% de la población sufre de esterilidad irreversible.
»Informo que en la próxima entrega de esperma y óvulos, que ocurre normalmente cada cinco décadas, que nos sirve para rejuvenecer la especie, no se podrá realizar, a menos que se encuentre a ese 0,1%. Caso contrario, nos iremos extinguiendo poco a poco. [...] Hace algunos años nuestro Instituto demostró que la vida de los clones se inicia a la edad del donante. Por ejemplo, cualquier Operador tiene una existencia útil de cuarenta años, sí se lo clona, la vida de este primer clon parte de cuarenta y cinco años. El clon de este, partirá de noventa años. El tercero, ciento treinta y cinco; y el cuarto, ciento ochenta. [...] Por consiguiente, habría una impresionante masa de Operadores Improductivos; y el sistema colapsaría.

**Supplementary Excerpt 5.** Corresponds to Section 4.1.2 of the main text (Londoño-Proaño, 2025b, pp. 32–33).

Pensó que no sería nada raro que ese empleado bursátil se declarara culpable de haber ingerido cantidades no permitidas de Boxín. Los Detectores mostrarían una sonrisa compasiva y le dirían que no se preocupara, que sucedía con frecuencia, que no había cometido ningún delito. «Como usted debe conocerlo, Operador 349», le dirían los Detectores, «el Boxín es una sustancia de consumo libre». El Operador respiraría aliviado y pediría disculpas por la impertinencia.
En su pantalla holográfica titiló un icono, indicándole que un bloque de acciones se había colocado a la venta en el mercado virtual. Verificó la procedencia. Era un paquete puesto por un Operador de la zona Oeste. Apenas eran diez acciones de uno de los laboratorios que elaboran Boxín. Le pareció que sus ruegos habían sido escuchados.
Esos laboratorios se revalorizan a diario, se dijo, elaboran un producto genético de consumo masivo. La misma Corporación Global lo distribuye gratuitamente en dosis de veinte ampolletas mensuales...

## 1.2 Quipucamayoc (Salvo, 2014)

**Supplementary Excerpt 6.** Corresponds to Section 4.2.1 of the main text (Salvo, 2014, p. 165).

“Une esos dos quipus. Así, muy bien. Ahora, anuda el último fleco, pero mostrando el nudo hacia ti. ¿Notas algo? Nada... bien. Ahora, haz lo mismo, pero con el nudo vuelto al revés. ¡Ah, una señal! ¿Qué te dice? ¿Que unas dos líneas sin nudo? ¿Para qué será? Haz lo que te dicen los nudos... ¡Ja ja ja! Bueno, ahí tienes la cantidad de hijos que tuvo realmente el anterior Inca. ¿Te parecen tantos? Observa bien el quipu, ahí hay un espacio vacío... cualquier nudo te sirve para saber que, tan solo en el transcurso de un año, casi todos esos niños murieron. ¡Ja ja ja! De esas cosas es mejor no hablar, joven aprendiz...” [...] Con orgullo, Pomacha pronto descubrió que sabía más acerca de la verdadera historia de los incas que los mismísimos autoproclamados hijos del Sol.

**Supplementary Excerpt 7.** Corresponds to Section 4.2.1 of the main text (Salvo, 2014, pp. 169–171).

—[...] Lo que dice un quipu puede interpretarlo cualquier quipucamayoc, quien a su vez puede modificar lo que está en el quipu. Un quipucamayoc no solo registra. Puede corregir. Puede cambiar. [...]
—Un quipucamayoc pone su marca en cada quipu que anuda. Un quipu pertenece a su quipucamayoc, y si es modificado por otro quipucamayoc, la modificación no altera la marca de origen, aunque esto solo vale para cinco modificaciones. [...]
—Mi señor, algún quipucamayoc muy sabio tejió en algún sitio y quien sabe hace cuánto tiempo los nudos malos. Nudos que introdujo en un quipu, se repitieron en otro, luego en otro, se volvieron a encontrar... [...]
—Los nudos malos tienen una señal que les dice a los quipucamayocs que los deben repetir en todos los quipus que anuden. Se repiten y se repiten, parece que desde hace años... Y no hay forma de corregirlos. El nudo malo que se desata de un quipu ya se anudó en otro.

**Supplementary Excerpt 8.** Corresponds to Section 4.2.1 of the main text (Salvo, 2014, pp. 171–172).

—Mi señor, ya no hay nada que hacer. Todos los quipus del imperio que se han usado para llevar informes de un lugar a otro no sirven más. El imperio ha perdido su lengua, sus palabras, sus pensamientos. Tendríamos que quemar todos los quipus y empezar a anudar otros nuevos... [...] No había entendido gran cosa de lo que le había informado el quipucamayoc, pero si algo estaba claro era que el imperio de los incas carecía de toda la información que hasta el momento lo había hecho funcionar: ya no existían los datos registrados sobre el poderío militar de los reinos conquistados, no había conocimiento de las cosechas recientes, no se podía saber cuánta gente había muerto y cuántos habían nacido en los últimos años. En suma, uno de los principales instrumentos de poder de los incas, que había servido eficientemente durante años para mantener el predominio de los hijos del Sol sobre otros pueblos, ya no existía.

**Supplementary Excerpt 9.** Corresponds to Section 4.2.1 of the main text (Salvo, 2014, pp. 172–173).

En el palacio del Cerro Azul, un envejecido y moribundo Guacro Cápac contemplaba el atardecer a las orillas del mar. Con una alegría poco disimulada, recibió las noticias de la exitosa rebelión que había culminado con el apresamiento y muerte de Huáscar y la entronización de su hermano Atahualpa como nuevo Inca. El poderío de los hijos del Sol estaba resquebrajándose. [...] Y pensar que todo se lo debía a Kuntur Ñahui y sus nudos malos, algo que nunca se tenía que hacer...

**Supplementary Excerpt 10.** Corresponds to Section 4.2.2 of the main text (Salvo, 2014, pp. 160–161).

Pero ser un quipucamayoc... Los quipucamayocs registraban la información que alimentaba al Tawantinsuyo. Gracias a los quipus, los incas habían alcanzado su actual supremacía sobre los demás pueblos, pues permitían almacenar información que se remontaba a períodos que el vulgo consideraba míticos. Guerras, sequías, hambrunas, viajes, depósitos, cultivos: toda esa información podía almacenarse en un quipu y ser descifrada por cualquier quipucamayoc. No era un conocimiento accesible a todos. No debía serlo. Kuntur Ñahui recordó como ejemplo el quipu que registraba la atroz muerte de las gentes de otros pueblos que sabían burilar signos en pallares, signos que decían cosas, no como las huacas o los quipus, pero con mayor certeza y claridad. Juzgó sabio al inca que había ordenado acabar con aquellos que podían guardar una verdad perenne distinta a la verdad registrada por los incas.

## 1.3 Yachak (Páez, 2003)

**Supplementary Excerpt 11.** Corresponds to Section 4.3.1 of the main text (Páez, 2007).

Los diminutos TSKZZ de Orkyyun, pasajeros de la asustada nave viviente, eran seres en continua mutación. [...] Su sociedad era una de las más exactas y programadas del Universo. Eran expertos constructores de máquinas que, dotadas con las características de la vida, mezclaban células, moléculas, unidades electrónicas y fluidos subatómicos. Entregaban el control de su existencia a sus creaciones, cuyas mentes seudoartificiales gobernaban su sociedad, sus viajes comerciales, sus tratados de intercambio tecno–económico con otras civilizaciones de la galaxia y su reproducción telepático genética. En el universo de los TSKSS todo estaba controlado, cronometrado, medido. Todo, hasta que EL MIEDO los dominaba. [...] Cuando EL MIEDO las atacaba nada podía salvarlos.

**Supplementary Excerpt 12.** Corresponds to Section 4.3.1 of the main text (Páez, 2007).

Sobre un mantel blanco, el Yachak había dispuesto sus piedras en las posiciones exactas. Cada una estaba a la distancia debida de las demás, entre todas reproducían el mundo y las fuerzas que lo constituyen. [...] En el centro del mantel brillaba negrísima una punta de flecha de obsidiana, perfecta, aguzada, prehistórica. Eran las piedras del poder. Entre ellas el Yachak había puesto distintos objetos: trozos de hierro, cobre y madera, un transparente pedazo de cristal que reflejaba la flama de dos velas colocadas a derecha e izquierda, estampas de santos, un crucifijo de mármol, fotografías suyas en las que aparecía acompañado de sacerdotes o médicos. Frente al Shamán, en el lado opuesto del mantel, se clavaban en la tierra una vieja y herrumbrada bayoneta de las guerras de la independencia, dos lanzas de chonta adornadas con plumas multicolores [...]. Todas las energías del mundo se reproducían y concentraban en la mesa del Yachak, por eso podía curar, conocer, controlar…

**Supplementary Excerpt 13.** Corresponds to Section 4.3.1 of the main text (Páez, 2007).

La lluvia corrosiva limpió como un bálsamo los canales bioelectrónicos de la nave. La energía encontraba sus ámbitos y fluía por los adecuados circuitos subatómicos. Una infinita quietud se apoderó del extraterrestre y de sus compañeros. La palabra de[l] Yachak, como un calmante masaje, ordenaba los contactos de las computadoras y restablecía el manejo electrónico molecular de los datos.

**Supplementary Excerpt 14.** Corresponds to Section 4.3.2 of the main text (Páez, 2007).

–Has enfermado mi mundo –dijo el Yachak–, has enfermado a mi hijo.
–Perdónanos –respondió el piloto, casi desmayado en la sala de controles de su nave–, no acostumbramos dañar a otros seres vivos, pero ahora estamos enfermos y hemos transmitido la enfermedad a tu mundo.
–¡Vete!
–No puedo. Moriremos pronto.
–Y tu enfermedad se quedar[á] y acabar[á] con mi mundo.
–Sí.
Ambos guardaron silencio. El Yachak sentía el dolor del demonio de la piedra de colores; el piloto captaba el sufrimiento de ese mundo que, sin querer, destruiría.
–¿Quién eres? –preguntó el Yachak.
–Un viajero –respondió el demonio–. ¿Y tú?
–Un Yachak, un curador.
–Cúrame.
José Sánchez lo pensó intensamente. Esa era la única esperanza para la Tierra, para su hijo.
–¿Qué tienes?
–Miedo.
–¿Espanto?
–Sí.
